# Tardigrada of Ireland: a review of records and an updated checklist of species including a new addition to the Irish fauna

**DOI:** 10.3897/zookeys.616.8222

**Published:** 2016-09-12

**Authors:** Erica DeMilio, Colin Lawton, Nigel J. Marley

**Affiliations:** 1 Animal Ecology & Conservation Unit, Department of Zoology, School of Natural Sciences, Martin Ryan Institute, National University of Ireland Galway, Republic of Ireland; 2 Marine Biology & Ecology Research Centre, Plymouth University, Drakes Circus, Plymouth, PL4 8AA, United Kingdom

**Keywords:** Tardigrada, Ireland, species list, historical records

## Abstract

The phylum Tardigrada was not recorded in Ireland until the Clare Island Survey of 1909–1911, with only rare subsequent reports on Irish tardigrade species. In recent decades, significant taxonomic revision has occurred within Tardigrada. This has resulted in the need for a review of all known historical records from Ireland and Northern Ireland in order to produce an updated checklist of valid taxa. The new checklist includes fifty-one tardigrade species and subspecies including a new addition to the Irish fauna reported herein, *Echiniscus quadrispinosus quadrispinosus* Richters, 1902 from Newtown, Ballyvaughan, Co. Clare.

## Introduction

Tardigrades, commonly known as “water bears”, are microscopic metazoans with body lengths typically between 0.1–1.2 mm. They are obligate aquatic organisms, occurring in marine, freshwater and damp terrestrial habitats such as soils, mosses and lichens. The body is cylindrical with four pairs of lobopodous appendages ending in claws or “toed” digits in many marine forms. Tardigrades are best known for the remarkable survival capabilities of many species through cryptobiosis, a type of quiescence that is also seen among other groups of microscopic animals found in similar habitats, notably Rotifera and Nematoda (for further information on tardigrade cryptobiosis see e.g. Guidetti et al. 2011, Møbjerg et al. 2011, Wełnicz et al. 2011).

Phylum Tardigrada has had a history of rearrangement, both in terms of its relative taxonomic position as well as its internal structure. Traditionally, Tardigrada was ranked as a class of Arthropoda until being recognised as a separate phylum by Ramazzotti (1962). Morphological and genetic analyses place the phylum within Ecdysozoa (Aguinaldo et al. 1997), most closely allied to Nematoda (e.g. Meusemann et al. 2010) or to Arthropoda with Onychophora as a sister group (e.g. Campbell et al. 2011). This phylogenetic relationship is, however, not considered completely resolved (Nielsen 2012, Borner et al. 2014).

The main intra-phylum division occurs between the classes Heterotardigrada Marcus, 1927 and Eutardigrada Richters, 1926. The heterotardigrades are a group of species that possess a particular cephalic structure known as cirrus A, they also have a separate gonopore and anus, and often have plated cuticles. Eutardigrade species lack cirrus A, have a cloaca, and cuticles without structures homologous to the sclerified plates present among heterotardigrades. A third monospecific class, Mesotardigrada, was described by Rahm (1937) from a Japanese hot spring. However, both the type specimen and locality are no longer extant and there is some doubt of the validity of the class (Nelson 2002).

The Heterotardigrada are divided into two orders, the marine Arthrotardigrada Marcus, 1927 and Echiniscoidea Richters, 1926. Echiniscoidea comprises four families: Echiniscoididae Kristensen & Hallas, 1980; Carphaniidae Binda & Kristensen, 1986; Oreellidae Puglia, 1959; and the speciose Echiniscidae Thulin, 1928. Of these, only Echiniscoididae is generally regarded as marine. Species of the other three families occur in limnic or limno-terrestrial environments.

There has been significant taxonomic rearrangement of the Eutardigrada including the establishment of many new genera and families, as well as many new species additions. Pilato (1969) contributed to this reorganization of Eutardigrada with the recognition of four familial lineages, Calohypsibiidae and Hypsibiidae (with subfamilies Diphasconinae Dastych, 1992 and Hypsibiinae Pilato, 1969); Milnesiidae Ramazzotti, 1962; and Macrobiotidae Thulin, 1928. Not in total agreement with the ranking of these families, Schuster et al. (1980) later established the orders Apochela for Milnesiidae (on the basis of the unique cephalic papillae and four separate and distinctive claws), and Parachela for all other eutardigrade families (in which cephalic papillae are not present and with the typical double claw formation).

Within Parachela, the use of morphological analyses and molecular data by Sands et al. (2008) and Marley et al. (2011) supported the creation of four superfamilies: Eohypsibioidea Bertolani & Kristensen, 1987 in Marley et al. 2011; Hypsibioidea Pilato, 1969 in Sands et al. (2008) (amended by Bertolani et al. 2014); Isohypsibioidea Sands, McInnes, Marley, Goodall-Copestake, Convey & Linse, 2008 (amended by Bertolani et al. 2014); and Macrobiotoidea Thulin, 1928 in Sands et al. (2008). The Parachela has undergone the most internal reshuffling at lower taxonomic levels, and species listed in early records may have been known under several names since.

The purpose of the following review is to address the taxonomic changes that have occurred for each Irish species since the time of their original recording. This review facilitated the creation of a valid checklist of Tardigrada species for Ireland and Northern Ireland.

## Review of existing irish literature

There has been very little investigation into the status of Tardigrada in Ireland. Studies that included Irish tardigrades are limited to: Murray (1911), Crisp and Hobart (1954), Le Gros (1959), Boaden (1966), Mitchell (1973), Morgan (1975, 1976 (includes unpublished data from Morgan’s doctoral thesis (1974) with further distribution notes from this study in Morgan and King 1976)), Baxter (1979), Morgan (1980), Kinchin (1990, 1992), Tumanov (2005) and Guidetti et al. (2015). Past work has been generally faunistic with little inference into ecology. Most species records for Ireland and Northern Ireland occurred prior to major taxonomic revisions within the phylum and so require clarification. The 2009 Inventory of Irish Fauna, by the Irish National Parks and Wildlife Service, included the Tardigrada (Smith 2009) but did not consider all literature pertaining to Irish tardigrades and only listed numerical values for species per family.

We updated the checklist of Irish species, which is presented in Table 1A–B, and have included a new record. The species list is in accordance with the latest version of the internationally recognised species list (Guidetti and Bertolani 2005, Degma and Guidetti 2007, Degma et al. 2015) and follows the amended classification for the Eutardigrada (Bertolani et al. 2014). Irish species that have not undergone alterations of their taxonomic position since their original recording in Ireland are not discussed in detail. Rather, the reader may be directed to the original sources for more information on these taxa.

**Table 1.** **A–B** An updated checklist of Irish tardigrade species with primary and subsequent records for: **A** Heterotardigrada **B** Eutardigrada. * Indicates type specimen. 1A Class Heterotardigrada Marcus, 1927

Species	Original Irish Record	Additional Irish records
**Order Arthrotardigrada** Marcus, 1927		
**Family Batillipedidae** Ramazzotti, 1962		
*Batillipes mirus* Richters, 1909b	Boaden 1966, Strangford, Lough, Co. Down	0
*Batillipes phreaticus* Renaud-Debyser, 1959	Morgan 1980, Brittas Bay, Co. Wicklow	0
*Batillipes tubernatis* Pollock, 1971	Morgan 1980, Belmullet and Achill Island, Co. Mayo; Gowlaun, Co. Galway; Brittas Bay, Co. Wicklow	0
**Order Echiniscoidea** Richters, 1926		
**Family Echiniscoididae** Kristensen and Hallas, 1980		
*Echiniscoides* sp.	Murray 1911, Achill Island, Co. Mayo	0
Echiniscoides sigismundi cf. sigismundi	Crisp and Hobart 1954, Cos. Waterford, Cork, Kerry, Sligo, Leitrim, and Donegal	Morgan 1980
**Family Echiniscidae** Thulin, 1928		
*Bryodelphax parvulus* Thulin, 1928	Murray 1911, Castlebar, Co. Mayo	0
*Cornechiniscus cornutus* (Richters, 1907)	Le Gros 1959, Kilsallah, Co. Mayo	0
Echiniscus columinis* Murray, 1911	Murray 1911, Achill Island, Co. Mayo	0
*Echiniscus granulatus* (Doyère, 1840)	Murray 1911, Castlebar, Co. Mayo	Le Gros 1959
Echiniscus militaris* Murray, 1911	Murray 1911, Castlebar, Co. Mayo	0
*Echiniscus quadrispinosus quadrispinosus* Richters, 1902	Present study, Newtown, Ballyvaughan, Co. Clare	0
*Echiniscus testudo* (Doyère, 1840)	Murray 1911, Castlebar, Co. Mayo	Morgan 1976
*Echiniscus trisetosus* Cuénot, 1932	Murray 1911, Castlebar, Co. Mayo	0
Pseudechiniscus cf. suillus	Murray 1911, Achill Island; Inishturk; Belclare, Co. Mayo	0
*Hypechiniscus gladiator gladiator* (Murray, 1905a)	Murray 1911, Achill Island, Co. Mayo	0
*Hypechiniscus exarmatus* (Murray, 1907a)	Murray 1911, Achill Island; Clare Island; Inishturk; Belclare, Co. Mayo	0
**Total = 16 taxa**		

**Table 1.** Continue. 1B Class Eutardigrada Richters, 1926

Species	Original Irish Record	Additional Irish records
**Order Apochela** Schuster, Nelson, Grigarick, and Christenberry, 1980		
**Family Milnesiidae** Ramazzotti, 1962		
*Milnesium* sp.	Murray 1911, Achill Island; Louisburgh; Westport; Castlebar, Co. Mayo	0
*Milnesium* cf. *tardigradumtardigradum*	Morgan 1976, “Galway”	Baxter 1979 Kinchin 1990
**Order Parachela** Schuster, Nelson, Grigarick, and Christenberry, 1980		
**Superfamily Hypsibioidea** Pilato, 1969 in Sands, McInnes, Marley, Goodall-Copestake, Convey, and Linse, 2008 (amended by Bertolani et al. 2014)		
**Family Calohypsibiidae** Pilato, 1969		
*Calohypsibius ornatus* (Richters, 1900)	Murray 1911, Achill Island; Clare Island; Louisburgh, Co. Mayo	0
*Calohypsibius verrucosus* (Richters, 1900)	Murray 1911, Clare Island, Co. Mayo	0
**Family Hypsibiidae** Pilato, 1969		
**Subfamily Diphasconinae** Dastych, 1992		
Diphascon cf. chilenense	Murray 1911, Clare Island, Co. Mayo	0
Diphascon cf. pingue	Mitchell 1973, Avoca, Co. Wicklow	0
**Subfamily Hypsibiinae** Pilato, 1969		
*Hypsibius arcticus* (Murray, 1907b)	Murray 1911, Clare Island; Inishturk; Louisburgh, Co. Mayo	0
Hypsibius cf. dujardini	Murray 1911, Achill Island, Co. Mayo	Morgan 1975, Baxter 1979 Kinchin 1990
**Subfamily Itaquasconinae** Bartoš in Rudescu, 1964		
*Adropion scoticum scoticum* (Murray, 1905b)	Murray 1911, Achill Island; Clare Island; Belclare, Co. Mayo.	Baxter1979
*Mesocrista spitzbergensis* (Richters, 1903)	Le Gros 1959, Kilsallah, Co. Mayo	0
*Platicrista angustata* (Murray, 1905a)	Murray 1911, Achill Island; Belclare, Co. Mayo	0
**Subfamily Pilatobiinae** Bertolani, Guidetti, Marchioro, Altiero, Rebecchi and Cesari, 2014		
*Pilatobius bullatus* (Murray, 1905b)	Morgan 1975, Termoncarragh, Co. Mayo	0
*Pilatobius oculatus oculatus* (Murray, 1906b)	Baxter 1979, Crawfordsburn; Helen’s Bay, Co. Down	0
**Family Microhypsibiidae** Pilato, 1998		
*Fractonotus caelatus* (Marcus, 1928)	Murray 1911, Clare Island Survey (precise location not given)	0
*Microhypsibius truncatus* Thulin, 1928	Morgan 1975, Annagh Head, Co. Mayo	0
**Family Ramazzottiidae** Sands, McInnes, Marley, Goodall-Copestake, Convey, and Linse, 2008		
*Hebesuncus conjungens* (Thulin, 1911)	Morgan 1975, Belmullet, Co. Mayo	0
**Superfamily Isohypsibioidea** Sands, McInnes, Marley, Goodall-Copestake, Convey and Linse, 2008 (amended by Bertolani et al. 2014)		
**Family Isohypsibiidae** Sands, McInnes, Marley, Goodall-Copestake, Convey and Linse, 2008		
*Isohypsibius annulatus annulatus* (Murray, 1905a)	Murray 1911, Clare Island and Castlebar, Co. Mayo	0
Isohypsibius panovi* Tumanov, 2005	Tumanov 2005, Bellharbour, Co. Clare	0
*Isohypsibius papillifer bulbosus* (Marcus, 1928)	Murray 1911 Clare Island, Co Mayo	0
*Isohypsibius prosostomus prosostomus* Thulin, 1928	Morgan 1975, Belmullet, Co. Mayo	Morgan 1976
*Isohypsibius prosostomus cambrensis* (Morgan, 1976)	Morgan 1976, Belmullet, Co. Mayo	0
*Isohypsibius schaudinni* (Richters, 1909b)	Murray 1911, Achill Island; Westport, Co. Mayo	Morgan 1975
*Isohypsibius tuberculatus* (Plate, 1888)	Murray 1911, Belclare; Castlebar, Co. Mayo	Baxter 1979
*Thulinius augusti* (Murray, 1907a)	Murray 1911 Louisburgh, Co. Mayo	0
**Superfamily Macrobiotoidea** Thulin, 1928 in Sands, McInnes, Marley, Goodall-Copestake, Convey and Linse, 2008		
**Family Macrobiotidae** Thulin, 1928		
*Macrobiotus crenulatus* Richters, 1904c	Murray 1911, Achill Island; Clare Island, Co. Mayo	0
*Macrobiotus echinogenitus* Richters, 1903	Murray 1911, Achill Island; Louisburgh; Belclare, Co. Mayo	0
Macrobiotus cf. harmsworthi	Murray 1911, Achill Island; Inishturk; Westport, Co. Mayo	Morgan 1975 Baxter 1979
Macrobiotus cf. hufelandi	Murray 1911, Achill Island; Clare Island; Inishturk; Louisburgh, Belclare; Westport; Castlebar, Co. Mayo	Le Gros 1959 Morgan 1975 Morgan 1976 Baxter 1979
*Macrobiotus occidentalis occidentalis* Murray, 1910	Murray 1911, Westport, Co. Mayo	0
*Macrobiotus virgatus* Murray, 1910	Murray 1911, Achill Island, Co. Mavo	0
Minibiotus cf. intermedius	Murray 1911 Achill Island; Clare Island; Inishturk; Belclare; Castlebar, Co. Mayo	Morgan 1975 Baxter 1979
*Paramacrobiotus areolatus* (Murray, 1907b)	Murray 1911 Achill Island, Co. Mayo	Morgan 1976
Paramacrobiotus richtersi* (Murray, 1911)	Murray 1911, Clare Island, Co. Mayo	Le Gros 1959 Morgan 1975 Morgan 1976
**Family Murrayidae** Guidetti, Rebecchi and Bertolani, 2000		
*Murrayon hastatus* (Murray, 1907a)	Murray 1911, Achill Island, Co. Mayo	0
Murrayon hibernicus* (Murray, 1911)	Murray 1911, Achill Island	0
**Total = 35 taxa**		

## James Murray and the Clare Island survey

The study of Irish tardigrades began with the work of the Scottish biologist and explorer, James Murray, as part of the multidisciplinary survey of Clare Island located off the west coast of County Mayo, Ireland (Murray 1911). The Clare Island Survey, 1909–1911, carried out by an international team of leading naturalists and scholars, aimed to describe the natural and archaeological history of the island. Murray completed two separate chapters on bdelloid rotifers and tardigrades. The great majority (approx. 70%) of the known Irish species to date are still those recorded by Murray (1911). In this initial survey thirty-five species of tardigrade were collected from thirteen sampling points on Clare Island and other nearby locations on mainland County Mayo. Four of these were new species. Unfortunately, while some of Murray’s other collections survive (see van der Land 1966, Greaves 1996, Dastych et al. 1998), his Clare Island material has not, and so re-examination of his collected specimens is not possible.

Murray (1911) collected both heterotardigrades and eutardigrades during the survey. All ten heterotardigrades recorded are Echiniscoidea (Table 1A). The only marine species was *Echiniscoides sigismundi* M. Schultze, 1865 (Echiniscoididae). Murray noted that his single specimen, obtained from sediment washed from seaweed, was morphologically different from both the description of *Echiniscoides sigismundi* by Schultze (1865) and the material of Richters (1909a). Once considered to be a single cosmopolitan species, Kristensen and Hallas (1980) recognized *Echiniscoides sigismundi* as a species complex that shows variation particularly in cuticular structure, claws, and gamete morphology over geographical area. Compared to the amended definition of *Echiniscoides sigismundi* *sensu stricto* by Kristensen and Hallas (1980), Murray’s specimen differed in claw arrangement with seven claws on leg pair IV, as opposed to the typical 8–10 claws on pair IV. This claw configuration can indicate *Echiniscoides sigismundi groenlandicus* Kristensen and Hallas, 1980 but Murray (1911) also noted that his specimen had unusually large spines (25 µm length) at the cirrus A position, large sense organs on leg pair four, and translucent papillae on the dorsum and body sides. This combination of features set Murray’s specimen apart from all known subspecies of *Echiniscoides sigismundi*. As the original specimen cannot be re-examined, a more detailed diagnosis cannot be made, and this record should be added to the Irish checklist as *Echiniscoides* sp.

The nine other heterotardigrade species reported by Murray (1911) are Echiniscidae (see Kristensen 1987 for a review of the family), two of which were new species. The Clare Island report includes Murray’s description of *Echiniscus militaris* Murray, 1911 from moss collected at a lakeshore in Castlebar, mainland Co. Mayo. The other new heterotardigrade, *Echiniscus columinis* Murray, 1911 was collected from the summit of Slievemore, a 671m mountain on Achill Island. In addition to the type, Murray also reported three forms that he suspected were related to *Echiniscus columinis*, but differed from the new species in lacking one or more of the lateral filaments and having different lengths of the filament in the Cd position. *Echiniscus militaris* and *Echiniscus columinis* have not undergone any taxonomic change, and are included in our Irish checklist as originally described by Murray.

Murray (1911) recorded *Echiniscus testudo* (Doyère, 1840) from Castlebar, Co. Mayo. He noted that the Irish *Echiniscus testudo* lacked filament B and exhibited a finer granulation than original figures of the type, yet the original text describing the type population stated that most specimens also lacked filament B. This species has been reported as showing variation in the arrangement of the lateral filaments (Ramazzotti and Maucci 1983). This variation in appendages reported by Murray for *Echiniscus columinis* and *Echiniscus testudo* is common among *Echiniscus* species, such as those in the *Echiniscus blumi-canadensis* series (Guil 2008). The difficulties in identifying individuals of such species is further complicated by the lack of supporting genetic data for the delineation of species in these series (Guil and Giribet 2009). There is a possibility that similar results might be seen in other *Echiniscus* species groups. Murray’s record for *Echiniscus testudo* is included in our list without any reference to the arrangement of the lateral appendages as the varieties ‘*trifilis*’ (lacking lateral B) and ‘*quadrifilis*’ (with lateral B) are not considered valid subspecies.

Other heterotardigrades that were recorded by Murray (1911) as *Echiniscus* C. A. S. Schultze, 1840 species were later moved to other genera of Echiniscidae mainly as a result of differences in the configuration of the dorsal cuticular plates. Murray recorded *Echiniscus suillus* (Ehrenberg, 1853) from four Mayo locations (two on Achill Island, two on the mainland). The genus *Pseudechiniscus* was erected by Thulin (1911) and more recently emended by Kristensen (1987). The species *suillus* was moved to this genus, which contains many morphologically similar species including the *Pseudechiniscus suillus* complex. The species in this complex can be very difficult to identify, even using modern criteria (Fontoura and Morais 2011). *Pseudechiniscus* has recently been included in an integrative taxonomic study by Vecchi et al. (in press). Their morphological and molecular data provided evidence for emending *Pseudechiniscus*, and the movement of some species (not *suillus*) into a new genus. Murray (1911) provided no notes for his record of this species, so the exact identity remains unclear and should be added to the Irish checklist as Pseudechiniscus cf. suillus.

Murray (1911) recorded *Echiniscus gladiator* (Murray, 1905a) and its variety *exarmatus* (Murray, 1907a). A single individual of the type was collected from Achill Island while *exarmatus* was noted as abundant among three sampling sites, including Clare Island. Thulin (1928) described the genus *Hypechiniscus* into which he moved both *Echiniscus gladiator* and the *exarmatus* variety. Kristensen (1987) favoured species rank for *exarmatus* as a result of its dissimilar claw morphology, and this ranking is now accepted (Guidetti and Bertolani 2005). These two records are included in the Irish checklist as *Hypechiniscus exarmatus* and *Hypechiniscus gladiator gladiator* in order to specify the type from three subspecies described by Iharos (1973).

The last three heterotardigrade records by Murray (1911) from the Clare Island Survey underwent later re-identification by Marcus (1936). Based on the literature, Marcus (1936) moved all Murray’s records for *Echiniscus granulatus* (Doyère, 1840) to *Echiniscus trisetosus* Cuénot, 1932, and synonymised *Echiniscus crassus* Richters, 1904a with *Echiniscus granulatus*. Also based on literature, Marcus (1936) deemed Murray’s northern hemisphere specimen of *Echiniscus intermedius* Murray, 1910 (=*Bryochoerus intermedius intermedius*) to be Echiniscus (Bryodelphax) parvulus (Thulin, 1928). While Thulin (1928) had previously erected the genus *Bryodelphax* for the species *parvulus*, Marcus (1936) only recognized *Bryodelphax* as a subgenus and continued to refer to the species as Echiniscus (Bryodelphax) parvulus. Both *Bryodelphax* and *Hypechiniscus*, remained as *Echiniscus* subgenera until re-elevated to genera (see: Ramazzotti and Maucci 1983; Kristensen 1987). Using Marcus’ (1936) interpretation for the three species identified by Murray (1911), we include, *Echiniscus trisetosus*, *Echiniscus granulatus* and *Bryodelphax parvulus* in the Irish checklist.

Murray recorded twenty-five eutardigrade species in the Clare Island Survey (Table 1B). Within Apochela, for over 150 years the genus *Milnesium* Doyère, 1840 was considered monospecific, despite a large degree of morphological variation observed across the highly cosmopolitan distribution of *Milnesium tardigradum*. As a result of the newly recognized diversity within the genus (e.g. Tumanov 2006, Michalczyk et al. 2012a), historical records for ‘*Milnesium tardigradum*’ that lack notes on taxonomic features currently in use for species identification, require further confirmation. Murray (1911) states that the Irish *Milnesium tardigradum*, collected from four separate sampling sites, had three points on each of the secondary claws of all legs (i.e. a claw formula of [3-3]–[3-3]). With great foresight, Murray recognized that it would be important to note this, as he suspected that the variation in claw morphology in *Milnesium* might subsequently be used in the delineation of new taxa; though he was likely thinking in terms of distinguishing local varieties rather than distinct species. Unfortunately, no further details for the *Milnesium* specimens were included in his description, yet on the basis of the claw configuration it can be concluded that Murray’s Irish specimens are not *Milnesium tardigradum* *sensu stricto* or its subspecies and so this record must be listed in our Irish checklist as *Milnesium* sp. sensu Michalczyk et al. (2012a, 2012b) and Morek et al. (2016).

The rest of Murray’s eutardigrade records are for Parachela. Following the convention of the time, Murray ascribed these species to only two long-standing genera, *Diphascon* Plate, 1888, differentiated by the presence of a flexible pharyngeal tube, or *Macrobiotus* C.A.S. Schultze, 1834. However, under the most current taxonomic scheme (Guidetti and Bertolani 2005, Degma and Guidetti 2007, Degma et al. 2015) including the amendments of Bertolani et al. (2014), twelve parachelan genera are represented in Murray’s (1911) Clare Island collection (Table 1B).

The specimens recorded by Murray (1911) as *Diphascon* species were: *Diphascon angustatum* Murray, 1905a, *Diphascon chilenense* Plate, 1888 and *Diphascon scoticum* Murray, 1905b. This genus has been considerably revised in recent years, and is now placed within the family Hypsibiidae Pilato, 1969, with a number of subfamilies including: Diphasconinae Dastych, 1992 and Itaquasconinae Bartoš in Rudescu, 1964, into which Murray’s (1911) taxa fall. Pilato (1987) recognized major divergence in the details of the buccal-pharyngeal apparatus and separated three additional genera from *Diphascon*: *Hebesuncus*, *Mesocrista*, and *Platicrista*. The two subgenera (from Pilato 1987): *Diphascon* and *Adropion* have now been elevated to genera (Bertolani et al. 2014). From Murray’s (1911) work, *Diphascon chilenense* now comes under the Diphasconinae and the genus name remains unchanged. However, this species is a member of the “*alpinum-pingue* group” and it is important to note that the lack of detail in original species descriptions for this group has made later identifications difficult. Several authors have discussed these difficulties (e.g. Dastych 1984, McInnes 1995, and Pilato and Binda 1977, 1998, 1999), with Pilato and Binda (1977, 1998) re-describing members of this group (*Diphascon alpinum* Murray, 1906a, *Diphascon chilenense*, *Diphascon pingue* and *Diphascon pinguiforme* Pilato and Binda, 1998). As Murray (1911) did not provide notes on his Irish *Diphascon chilenense* material we cannot interpret the correct species diagnoses. It is possible the Clare Island *Diphascon chilenense*, collected only from the summit of Croaghmore [Knockmore] (462m), was a similar species within the species-group. Murray’s (1905a) “*Diphascon angustatum*” was used by Pilato (1987) to erect the genus *Platicrista*, within the subfamily Itaquasconinae, and is the genus type. Therefore, Murray’s (1911) reference is now, with the corrected suffix, *Platicrista angustata*. The third species “*Diphascon scoticum*” Murray, 1905b has become the genus type for *Adropion*, and is now *Adropion scoticum scoticum*. As a result of the above amendments, we are modifying Murray’s (1911) *Diphascon* records for the Irish checklist to be: Diphascon cf. chilenense, *Adropion scoticum scoticum* and *Platicrista angustata*.

Of the twenty-two other species attributed to *Macrobiotus* that were recorded by Murray (1911) for the Clare Island Survey, only six remain in that genus today. Some of these taxa have been repositioned several times. Four of Murray’s (1911) records were: all three of Richters’ (1900) *Macrobiotus ornatus* Richters, 1900 varieties (i.e. *spinnifer*, *spinosissimus*, and *verrucosus*) from western Ireland (Murray 1911), and *Macrobiotus scabrosus* Murray, 1911, which Murray (1911) described from Clare Island itself. These species have been associated to the genera *Calohypsibius* Thulin, 1928 and *Microhypsibius* Thulin, 1928. Initially, Thulin (1911) re-instated *Hypsibius* Ehrenberg, 1848 and moved both “*Macrobiotus ornatus*” and “*Macrobiotus scabrosus*” into this genus, and raised “*Macrobiotus ornatus* v. *verrucosus*” to species rank (“*Hypsibius verrucosus*”). Upon further consideration of the type material, Thulin (1928) erected the genus, *Calohypsibius*, with *Calohypsibius ornatus* as the genus type, into which the species “*Hypsibius ornatus*”, “*Hypsibius scabrosus*”, and “*Hypsibius verrucosus*” were moved. According to Marcus (1936) *Calohypsibius* was a sub-genus and as Richters’ (1900) failed to designate which of the three described varieties of *ornatus* was the type specimen, nominated *spinnifer* for this position. More recently, Pilato (1969) upheld the validity of *Calohypsibius* as a genus, and proposed its placement into a new family, Calohypsibiidae Pilato, 1969 (amended by Bertolani et al. 2014). Pilato (1989) and Pilato et al. (1989) further discussed the wide variation of morphologies reported for *Calohypsibius ornatus*, and the associated varieties, and the resulting need for taxonomic revision of the species. Pilato (1998) erected the genus *Fractonotus* (in the family Microhypsibiidae Pilato, 1998) for the species *caelatus*, as the claws were determined to be closer to that of *Microhypsibius* Thulin, 1928 than of *Calohypsibius* Thulin, 1928. From Murray’s (1911) list, in Marcus’ (1936) opinion Murray misinterpreted “*Macrobiotus ornatus* v. *verrucosus*”, which he moved to “Hypsibius (Calohypsibius) ornatus v. *caelata* Marcus, 1928”. This subspecies has subsequently been elevated to species and moved to the genus *Fractonotus*. Marcus (1936) also concluded from the literature that Murray’s (1911) species “*scabrosus*” was actually “*Macrobiotus ornatus* v. *verrucosus*” moving it to “Hypsibius (Calohypsibius) verrucosus”. Following these revisions, we are adding Murray’s (1911) records for these taxa to the Irish checklist as: *Calohypsibius ornatus* (Richters, 1900) (from “Macrobiotus ornatus var. spinnifer” and “*Macrobiotus ornatus* v. *spinosissimus*” (no longer considered valid subspecies: see: Bartoš, 1940)); *Calohypsibius verrucosus* (Richters, 1900) (from “*Macrobiotus scabrosus* sp. nov.”); and *Fractonotus caelatus* (Marcus, 1928).

Four of the “*Macrobiotus*” taxa Murray (1911) recorded have been moved into the genus *Isohypsibius* Thulin, 1928. These are: “*Macrobiotus annulatus* Murray, 1905a”; “*Macrobiotus schaudinni* Richters, 1909b”; “*Macrobiotus tuberculatus* Plate, 1888”; and “*Macrobiotus papillifer* Murray, 1905a”. Murray’s (1911) record of “*Macrobiotus papillifer*” from Clare Island was not for the type itself, but a variety that he had previously encountered in Scotland, though had not described. Marcus (1936) elevated this to subspecies but reduced the genus to subgenus: “Hypsibius (Isohypsibius) papillifer bulbosus Marcus, 1928”. *Isohypsibius* has been returned to generic ranking (Pilato 1969), and is now the most speciose genus of family Isohypsibiidae, Sands, McInnes, Marley, Goodall-Copestake, Convey, and Linse, 2008. We include in the Irish checklist the four taxa from Murray’s (1911) list as: *Isohypsibius papillifer bulbosus*, *Isohypsibius annulatus annulatus*, *Isohypsibius schaudinni* and *Isohypsibius tuberculatus*.

Another of Murray’s (1911) Clare Island ‘*Macrobiotus*’ records, that for “*Macrobiotus augusti* Murray, 1907a”, is also now in the family Isohypsibiidae. There has been much confusion about “*augusti*”, which is detailed in Bertolani et al. (1999) and Bertolani (2003). In summary, Thulin (1928) moved “*Macrobiotus augusti*” into the genus *Isohypsibius* and Marcus (1929) from the literature re-described the species as Hypsibius (Isohypsibius) augusti, adding characters that were not present in the type specimen. Subsequent use of erroneous re-descriptions of the species by later authors perpetuated the confusion (for details see also Marley et al. 2008). The genus *Pseudobiotus* Nelson, 1980 in Schuster et al. 1980, with the genus type *Pseudobiotus augusti*, was established upon such a re-description. Later re-examinations of “*Macrobiotus augusti*” type material led Nelson et al. (1999) to re-describe *Pseudobiotus* with the designation of a different type species, and Bertolani et al. (1999) to move “*augusti*” to the genus *Thulinia* Bertolani, 1982 (NB. Bertolani (2003) substituted the genus name with *Thulinius*, as *Thulinia* was already in use for a genus of trematodes). Thus, we are adding Murray’s (1911) record for “*Macrobiotus augusti*” to the Irish taxa as: *Thulinius augusti* (Murray, 1907a).

Two of Murray’s (1911) “*Macrobiotus*” records are now *Hypsibius* species. Murray (1911) recorded both “*Macrobiotus lacustris* Dujardin, 1851” and “*Macrobiotus arcticus* Murray, 1907b” from multiple Clare Island Survey sites. Thulin (1911, 1928) and Marcus (1936) agreed that *Macrobiotus lacustris* was synonymous with *Hypsibius dujardini* (Doyère 1840). This species is now known to be morphologically very similar to others in the *convergens-dujardini* complex (Miller et al. 2005, Kaczmarek and Michalczyk 2009). As Murray (1911) made no descriptive notes on his specimens we include this record in the Irish checklist as: Hypsibius cf. dujardini. The second species, “*Macrobiotus arcticus*”, was initially described by Murray (1907b) from juveniles and eggs collected from Prince Charles Foreland, Franz Josef Land and Loch Ness (Scotland), noting that the eggs were similar to “*Macrobiotus hastatus*”, but he later emended the description with details of adult animals and similar eggs found at Cape Royds (Antarctica) (Murray 1910). The later were *Acutuncus antarcticus* (Richters, 1904b) (see: Dastych 1991), leaving “*Macrobiotus arcticus*” with scant information and inadequate data for species identification (Dastych 1991). However, Thulin (1911) transferred “*arcticus*” into *Hypsibius*, and despite the shortcomings of the original description (see: Dastych 1991), the species is still valid. We include *Macrobiotus arcticus* in the Irish checklist as: *Hypsibius arcticus*.

The remaining nine species from Murray’s (1911) Clare Island list are all from the superfamily Macrobiotoidea. Only six now remain in the genus *Macrobiotus* as originally recorded: *Macrobiotus crenulatus* Richters, 1904c; *Macrobiotus harmsworthi* Murray, 1907b; *Macrobiotus hufelandi* C. A. S. Schultze, 1834; *Macrobiotus echinogenitus* Richters, 1903; *Macrobiotus occidentalis* Murray, 1910; and *Macrobiotus virgatus* Murray, 1910. *Macrobiotus hufelandi* and *Macrobiotus harmsworthi* are now known to represent large species-groups. It is notoriously difficult to differentiate between species within these groups, even with modern microscopy, and identification usually requires observation of the egg (Bertolani and Rebecchi 1993). Therefore, the records for these species can at present only be listed as Macrobiotus cf. hufelandi and Macrobiotus cf. harmsworthi. However, the position of the *Macrobiotus harmsworthi* group in the genus *Macrobiotus* will soon be revised as a result of new morphological and molecular analyses (Vecchi et al. in press). Murray’s (1911) records for *Macrobiotus crenulatus*, *Macrobiotus echinogenitus*, *Macrobiotus occidentalis*, and *Macrobiotus virgatus* remain unchanged in the Irish checklist. In addition to these records, Murray (1911) collected an egg with an embryonic tardigrade within. He was not able to identify this further than ‘*Macrobiotus* species’ and his notes and figures do not provide for a definitive conclusion. This record has been omitted from our Irish checklist.

Three of Murray’s (1911) records have been transferred from *Macrobiotus* into other Macrobiotidae genera. One of these, “*Macrobiotus intermedius* Plate, 1888”, became the type species for the genus *Minibiotus* differentiated by Schuster et al. 1980 from *Macrobiotus* by a lack of peribuccal lamellae and an enclosing egg membrane. Recognizing some difficulties with the original diagnoses, including the fact that not all *Minibiotus* eggs share this membrane, the genus and the species *intermedius* were re-described by Claxton (1998). Her study also discussed a species group within *Minibiotus* of morphologically similar adults that includes “*intermedius*”. As Murray (1911) did not provide morphological notes on his “*Macrobiotus intermedius*” specimens, this record must be listed as Minibiotus cf. intermedius.

The two other species, recorded from Clare Island itself, *Macrobiotus areolatus* Murray, 1907b and *Macrobiotus richtersi* Murray, 1911 (Murray 1911) have been moved into the genus, *Paramacrobiotus* by Guidetti et al. (2009) based upon combined morphological and molecular evidence. Murray’s (1911) “*richtersi*”, described from a salt marsh on Clare Island, was used as the type species for the new genus. In addition to the type, Murray (1911) also recorded a variety of “*richtersi*” from the mainland, which differed from the type mainly in the formation of the egg processes and in the relative lengths of the macroplacoids. This variety was not recorded in Ireland subsequently and we have omitted it from our Irish checklist. *Paramacrobiotus areolatus* and *Paramacrobiotus richtersi* are included, however integrative analyses by Guidetti et al. (2015) of *Paramacrobiotus richtersi* specimens from the type location on Clare Island and various Italian localities suggest the presence of a cryptic species complex, highlighting the potential importance of integrated taxonomy, incorporating alpha taxonomy and DNA-barcoding, in future identifications of this species.

The last two of Murray’s (1911) records, *Macrobiotus hastatus* Murray, 1907a and *Macrobiotus hibernicus* Murray, 1911, have been moved into the genus *Murrayon* Bertolani & Pilato, 1988, which was named in honour of James Murray. The “*hastatus*” species, described by Murray from material collected during the Scottish Loch Survey (Murray 1905a, 1907a) was moved into *Murrayon* at the time of the creation of the genus (Bertolani and Pilato 1988). The species “*hibernicus*”, which was described during the Clare Island survey from a tarn on Slievemore, Achill Island (Murray 1911), was moved later by Guidetti (1998) following a reanalysis of material from Italy and Greenland. A detailed comparison of the cuticular structure and claw formation of some genera of Macrobiotidae led Guidetti et al. (2000) to suggest the split of the family into two subfamilies, Macrobiotinae and Murrayinae with *Murrayon* as the type genus for the latter. Further phylogenetic analysis using morphological and molecular data by Guidetti et al. (2005) gave further support for the division of the Murrayinae line from other macrobiotid taxa. The group was then raised to family level (Murrayidae Guidetti, Rebecchi and Bertolani, 2000). Although recent molecular evidence suggests that Murrayidae may be polyphyletic, no morphological support has yet been found (Bertolani et al. 2014). We include Murray’s (1911) two species in the Irish Checklist as: *Murrayon hibernicus* and *Murrayon hastatus*.

## Records by later authors

Following Murray’s (1911) work for the original Clare Island Survey, the study of tardigrades was neglected in Ireland. No further references to Tardigrada in Ireland can be found in the literature until Crisp and Hobart’s (1954) investigation into the distribution of *Echiniscoides sigismundi* on Irish and British coasts. This paper (Crisp and Hobart 1954) was the first strictly ecological study on tardigrades in Ireland, which identified the host, zonation, and seasonal variables of *Echiniscoides sigismundi* at several sites around the Irish coast. *Echiniscoides sigismundi* was recorded from beaches in counties: Waterford, Cork, Kerry, Sligo, Leitrim, and Donegal, suggesting a widespread distribution of the species on Irish intertidal shores. The authors (Crisp and Hobart 1954) did not comment in detail on the morphology of Irish specimens beyond that these matched well with the original description. The differing morphology of the *Echiniscoides sigismundi* specimens of Murray (1911) to those of Crisp and Hobart (1954) supports the inclusion of two separate *Echiniscoides* records in the Irish checklist. As none of the known subspecies had been described at the time of Crisp and Hobart’s (1954) study and no variation was reported, it cannot be certain which subspecies they encountered and so is accounted for in the Irish the checklist as Echiniscoides sigismundi cf. sigismundi.

New species records for Ireland did not occur until Le Gros (1959), nearly fifty years after the Clare Island Survey. A small quantity of moss and lichen samples collected from Kilsallah, County Mayo, yielded five species of tardigrade, two of which had not been previously recorded. Le Gros’ (1959) new additions, *Pseudechiniscus cornutus* (Richters, 1907) and *Hypsibius spitzbergensis* (Richters, 1903) (originally described as *Diphascon spitzbergense*) were both later moved into new genera. *Pseudechiniscus cornutus* became the type species for the genus *Cornechiniscus* Maucci and Ramazzotti 1981 (revised by Kristensen (1987)), for those echiniscids belonging to what was then known as the ‘*Pseudechiniscus cornatus* group’ of species possessing distinctive cirri A in the form of short, recurved spines and with particular features of the cuticular plates. *Hypsibius spitzbergensis* (or *Diphascon spitzbergense*) became the species type for the genus *Mesocrista* Pilato, 1987. As a result, we include these records in the Irish checklist as: *Cornechiniscus cornutus* and *Mesocrista spitzbergensis*.

The heterotardigrade order, Arthrotardigrada, was not recorded from Ireland until Boaden’s (1966) investigation of the interstitial fauna of the area surrounding Strangford Lough in County Down, Northern Ireland. *Batillipes mirus* Richters 1909b (Batillipedidae Ramazzotti, 1962) was the only tardigrade species reported by Boaden (1966), who found an unspecified number of individuals among fine sand from the northern end of the lough. The record for *Batillipes mirus* was one among many other taxa from a variety of phyla, and there is no specific discussion of the tardigrade specimen.

Mitchell (1973) was next to make a new addition to the Irish fauna from samples of cherry tree bark from Avoca, Co. Wicklow. Mitchell (1973) recorded an unknown number of specimens, though certainly more than one, of ‘Hypsibius (Diphascon) pinguis’ (i.e. *Diphascon pingue*). The species was later considered to belong to genus Diphascon (subgenus Diphascon) until the aforementioned amendments to Diphasconinae by Bertolani et al. 2014. Le Gros is acknowledged as confirming Mitchell’s identification, though Mitchell (1973) noted that the Irish specimens have longer body lengths and narrower placoid rows than the original figures of Marcus (1936). There has been some uncertainty with the “*alpinum*-*pingue*” species group, as discussed above, and there is some possibility that Mitchell (1973) may have collected *Diphascon pinguiforme*. Mitchell’s (1973) notes on the width:length ratio of the pharyngeal bulb and larger body size are closer to the values given for the re-described *Diphascon pingue* (Pilato and Binda 1998). However, slide pressure can change these ratios and as there was no figure or mention of the drop-shaped thickening between the buccal and pharyngeal tubes this record must remain questionable. We include this record in the Irish checklist as Diphascon cf. pingue.

Along with Murray (1911), Clive Morgan has perhaps been the greatest contributor to the study of Tardigrada in Ireland. Morgan (1974) included Irish sites in a survey of the British Isles as part of his doctoral thesis research. These were given in Appendix II of Morgan’s (1974) thesis as “Belmullet Peninsula” (four sites) and “Galway Bay” (seven sites). The Galway Bay sites, were listed as: “Aran Island” (assumed to be Inishmore judging by Figure 1 in Morgan 1976), Ardfry, Galway [City], Spiddal, and Mweenish Island, Co. Galway, and Finavarra and Doolin Point, [Co. Clare]. Morgan’s (1974) thesis does not specify precisely which of the “Galway Bay” locations contained which species. His results (Morgan 1974; table 6) are for 14 taxa belonging to “Galway” (three species) or “Mayo” (eleven species and one variety). There is also an omission from the table corresponding to the notes on species distribution in the main text, as *Paramacrobiotus areolatus* (then *Macrobiotus areolatus*) was recorded from moss from Belmullet, Mayo but was not marked as present in the table’s column for Mayo.

**Figure 1.**
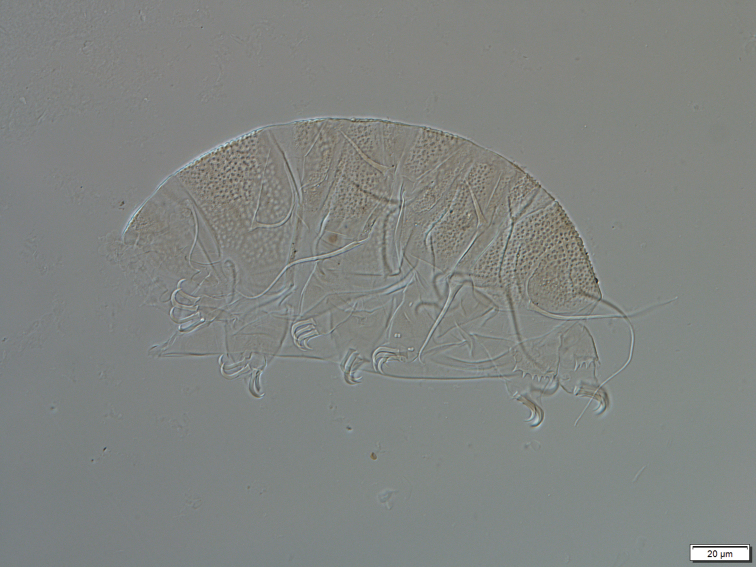
*Echiniscus quadrispinosus quadrispinosus* Richters, 1902, habitus.

Further details on species locations are given in two of Morgan’s subsequent works (Morgan 1975, 1976) in which the results from his thesis were published, and in Morgan and King’s (1976) “Synopsis of the British Fauna-Tardigrada”. Morgan (1975) states that the material for his notes on the Tardigrada from the Mullet Peninsula was obtained as part of the survey of the area conducted by the University of Reading (1971–1972), but this material was probably used as part of his doctoral research. Along with seven previously reported species, with details on sampling sites and a key to the Irish species, Morgan (1975) added four new records for Ireland (identified in his thesis (Morgan 1974)) as: Hypsibius (Diphascon) bullatus (Murray, 1905b); Hypsibius (Hypsibius) conjungens Thulin, 1911; Hypsibius (Isohypsibius) prosostomus (Thulin, 1928); and, Hypsibius (Calohypsibius) truncatus (Thulin, 1928). All these subgenera have been elevated to genera. Hypsibius (Diphascon) bullatus having a drop-shaped thickening on the bucco-pharyngeal tube was moved to Diphascon (Diphascon) bullatum with the amended suffix, but has since moved to the genus *Pilatobiu*s Bertolani, Guidetti, Marchioro, Altiero, Rebecchi & Cesari, 2014, and the suffix corrected back to ‘*bullatus*’. *Hypsibius conjungens* was used as the type species for the genus *Hebesuncus* Pilato 1987. Morgan’s (1975) record for Hypsibius (Isohypsibius) prosostomus has been elevated to the genus *Isohypsibius* and remains unchanged. The species “*truncatus*” had been the species type for the genus *Microhypsibius* Thulin 1928, but was suppressed by Marcus (1929) as Hypsibius (Calohypsibius) truncates. The genus *Microhypsibius* was later re-instated and re-described by Kristensen (1982) and since moved (along with the genus, *Fractonotus*) into the family, Microhypsibiidae Pilato 1998. We therefore, include Morgan’s (1975) records in the Irish checklist as: *Pilatobius bullatus*, *Hebesuncus conjungens*, *Isohypsibius prosostomus prosostomus* and *Microhypsibius truncatus*.

Further results from Morgan’s earlier Irish collections were published in 1976 (Morgan 1976) along with data from mainland Britain and offshore islands. Only three species were recorded for “Galway” (Morgan 1976, table 1): *Macrobiotus hufelandi*, *Macrobiotus richtersi* (now *Paramacrobiotus*), and *Milnesium tardigradum*. However, a new Irish record is listed in the systemic account of species, which gives Belmullet, Co. Mayo as secondary location for the new subspecies Hypsibius (Isohypsibius) prosostomus cambrensis (now *Isohypsibius prosotomus cambrensis*), first described by Morgan (1976) from moss collected at the University of Swansea. Morgan (1976) described *Isohypsibius prosostomus cambrensis* as similar to the type but with fine cuticular granulation present on the sides of the body and upper portions of all legs. Pending a re-examination of the type material, the presence of this granulation may warrant the elevation of *Isohypsibius prosostomus cambrensis* to species rank. The only other reference from this survey to a specific Irish location is found in Morgan and King’s Synopsis of the British Fauna (1976) in which Mweenish Island (Co. Galway) is named in the distribution notes for *Milnesium tardigradum*. Morgan’s (1976) record for this species is included in our Irish checklist as Milnesium cf. tardigradum, as no morphological notes were provided.

More recently, Morgan (1980) sampled the marine habitat in Counties Galway, Mayo and Wicklow. Eighteen samples yielded three marine species, two of which had not previously been recorded in Ireland, *Batillipes phreaticus* Renaud Debyser, 1959 and *Batillipes tubernatis*, Pollock 1971. Irish *Batillipes phreaticus* and *Batillipes tubernatis* populations were reported to have some differences in the morphometric values for several appendage lengths compared with those of the type locations (Morgan 1980). Morgan (1980) also recorded *Echiniscoides sigismundi*, which showed some variation in punctuation of the cuticle between the Irish sampling locations but was reported to match well with the then current species descriptions. We include these in the Irish list as: *Batillipes phreaticus*, *Batillipes tubernatis*, and Echiniscoides sigismundi cf. sigismundi.

Baxter (1979) carried out sampling in north County Down, Northern Ireland, producing the first records of terrestrial tardigrades for the region. The mosses and lichens obtained there were found to contain eight species of tardigrade which Baxter (1979) recorded as: Hypsibius (Diphascon) oculatus (Murray, 1906b), Hypsibius (Diphascon) scoticus, Hypsibius (Hypsibius) dujardini, Hypsibius (Isohypsibius) tuberculatus, *Macrobiotus harmsworthi*, *Macrobiotus hufelandi*, *Minibiotus intermedius*, and *Milnesium tardigradum*. One of these records, Hypsibius (Diphascon) oculatus, was new for Ireland. Having a drop-shaped thickening on the bucco-pharyngeal tube, “*oculatus*” became Diphascon (Diphascon) oculatus before being transferred to *Pilatobius*. As an additional note (E.D. – personal notes and observations): representative material for each species collected by Baxter (1979) was deposited in the National Museum of Ireland - Natural History, Dublin, and was available for study. The specimens of the Baxter Collection are in general, not well-preserved. Some specimens are in better condition but are situated along the coverslip margins. These would require specialist long working distance lenses at higher magnifications for clear observation due to a raised lip of sealant. However, some additional observations of the specimens were possible.

Baxter (1979) recorded *Milnesium tardigradum* but presented no details of claw morphology. Upon examination of Baxter’s museum material, it was not possible to ascertain the claw formula for one specimen but, assuming all specimens on a single slide were the same species, the claw formula was [2-3]–[3-2] with accessory points present on the primary branches indicating Milnesium cf. tardigradum (in contrast to Murray (1911)). In the Baxter Collection are specimens recorded as ‘Hypsibius (Hypsibius) dujardini’ from two separate populations. The specimens of one population are morphologically similar to *Hypsibius dujardini* but the presence or absence of characters that separate this species from others in the *dujardini*-group could not be confirmed. The specimens from the other population appeared to have more granular macroplacoids than the species of the *dujardini* group. These were more similar in appearance to those of *Hypsibius microps* Thulin, 1928 or *Hypsibius pallidus* Thulin, 1911. It is possible that this population represents one of these species but this could not be confirmed. In addition to the eight species recorded, a single degraded specimen of an unidentified *Isohypsibius* species was observed among the Baxter Collection but was not included in the Irish checklist due to a lack of further information. Consequently we included the results of Baxter’s (1979) collection in the Irish checklist as: *Pilatobius oculatus oculatus* (as two other subspecies of *Pilatobius oculatus* are known (Murray 1910, Mihelčič 1964)), *Adropion scoticum scoticum*, Hypsibius cf. dujardini, *Isohypsibius tuberculatus*, Macrobiotus cf. harmsworthi, Macrobiotus cf. hufelandi, Minibiotus cf. intermedius, and Milnesium cf. tardigradum.

The most recent published data on Irish tardigrades are Kinchin (1990) and Tumanov (2005). Kinchin (1990) recorded *Milnesium tardigradum* and *Hypsibius dujardini* associated with lichens from the Giant’s Causeway, Co. Antrim, Northern Ireland. Morphological notes on the specimens were not provided so it was not possible to confirm these records. Kinchin (1992) recording microfauna from various locations in Britain and Ireland included his previous data from Co. Antrim, and reported Rotifera and Nematoda in moss and lichen from Dingle, Co. Kerry but found no Tardigrada. Tumanov (2005) described a new tardigrade species, *Isohypsibius panovi* from Bellharbour, County Clare.

## A new addition to the Irish fauna

### *Echiniscus quadrispinosus quadrispinosus* Richters, 1902

A specimen, found in a sample of moss collected from a tree trunk along a rural road in Newtown, Ballyvaughan, Co. Clare (53°6.28'N; 9°10.18'W) in January 2013, was a new record for Ireland and is added to our checklist. The specimen was mounted with Polyvinyl Alcohol medium and identified using an Olympus BX53 microscope with magnification up to x1000 oil immersion. All measurements were taken using Olympus cellSens imaging software (Standard Version 1 CS-ST-V1).

A single adult individual was found (Figure 1). Body length excluding fourth pair of legs, 174 µm. Double granulation present, clearly different from *Echiniscus merokensis* Richters, 1904c type. Scapular plate with small accessory plates. Terminal plate facetted, visible is a thin lateral band without granulation. Internal claws 10 µm long, with basal spurs. Fourth pair of legs bear dentate collar with eight well-separated teeth of irregular lengths. Lateral filaments; A 33 µm, B 28 µm, C 30 µm, D 38 µm, E 54 µm. Dorsal spines; Cd 24 µm, Dd 18 µm. Specimen laterally positioned, thus gonopore morphology is not discernible, sex unknown.
